# 25 Years of PI5P

**DOI:** 10.3389/fcell.2023.1272911

**Published:** 2023-10-02

**Authors:** Lucia E. Rameh, Raymond D. Blind

**Affiliations:** Department of Medicine, Division of Diabetes, Endocrinology and Metabolism, Vanderbilt University Medical Center, Nashville, TN, United States

**Keywords:** PI5P, PIP4K, PI(4,5)P_2_, PIKfyve, TMEMs

## Abstract

The accidental discovery of PI5P (phosphatidylinositol-5-phosphate) was published 25 years ago, when PIP5K type II (phosphoinositide-4-phosphate 5-kinase) was shown to actually be a 4-kinase that uses PI5P as a substrate to generate PI(4,5)P_2_. Consequently, PIP5K type II was renamed to PI5P4K, or PIP4K for short, and PI5P became the last of the 7 signaling phosphoinositides to be discovered. Much of what we know about PI5P comes from genetic studies of PIP4K, as the pathways for PI5P synthesis, the downstream targets of PI5P and how PI5P affects cellular function all remain largely enigmatic. Nevertheless, PI5P and PI5P-dependent PI(4,5)P_2_ synthesis have been clearly implicated in metabolic homeostasis and in diseases such as cancer. Here, we review the past 25 years of PI5P research, with particular emphasis on the impact this small signaling lipid has on human health.

## 1 An accidental discovery

The phosphoinositide PI5P was fortuitously discovered (Rameh et al., 1997) in control reactions for an *in vitro* lipid phosphatase activity assay in the Cantley Lab. The phosphatase being assayed was SHIP1, a 5-phosphatase. A commercially available PI4P prep from crude brain was used as substrate to generate PI(4,5)P_2_, labeled with ^32^P presumably at the 5-position of the inositol ring using the kinase formerly known as “PIP5K type II”. We now know this PI4P prep contained contaminating PI5P, which at the time was not known to exist in living cells. Regardless, PI(4,5)P_2_ was intended as a negative control in a SHIP1 activity assay, as this phosphatase was thought to specifically dephosphorylate PI(3,4,5)P_3_. To our surprise, SHIP1 dephosphorylated PI(4,5)P_2_ and furthermore, the monophosphorylated product retained the ^32^P radiolabel. If “PIP5K type II” had added the ^32^P label to the 5 position, the known 5-phosphtase activity of SHIP1 should have removed it. The puzzle was solved by comparing the ^32^P-PI(4,5)P_2_ labeled by “PIP5K type II” with the ^32^P-PI(4,5)P_2_ labelled by PIP5K type I, which showed PIP5K type I and type II were introducing the ^32^P at different positions of the inositol ring, and as we now know, were using different substrates (Rameh et al., 1997). We then postulated the existence of PI5P, which had never been reported. The challenge was to develop an HPLC method for separating the putative PI5P from other monophosphorylated phosphoinositides, especially PI4P, which is highly abundant in cells. This was accomplished by trial and error using variations on the ammonium phosphate gradient until a small shoulder in the chromatogram appeared out of the abundant PI4P peak. This small peak coincided with the product generated by the dephosphorylation of ^32^P-PI(4,5)P_2_ labeled by type I PIPK, but not the product of the PI(4,5)P_2_ labeled by type II. At that point the picture became clear: the type II PIP5K was actually using PI5P as a substrate, present as a contaminant in the commercially sold PI4P, revealing that the type II enzyme is a 4-kinase, not a 5-kinase as previously thought. “Type II PIP5K″ was then renamed PIP4K and its substrate preference was further confirmed using synthetic phosphoinositides, which themselves had just become available (Rameh et al., 1997).

**FIGURE 1:**
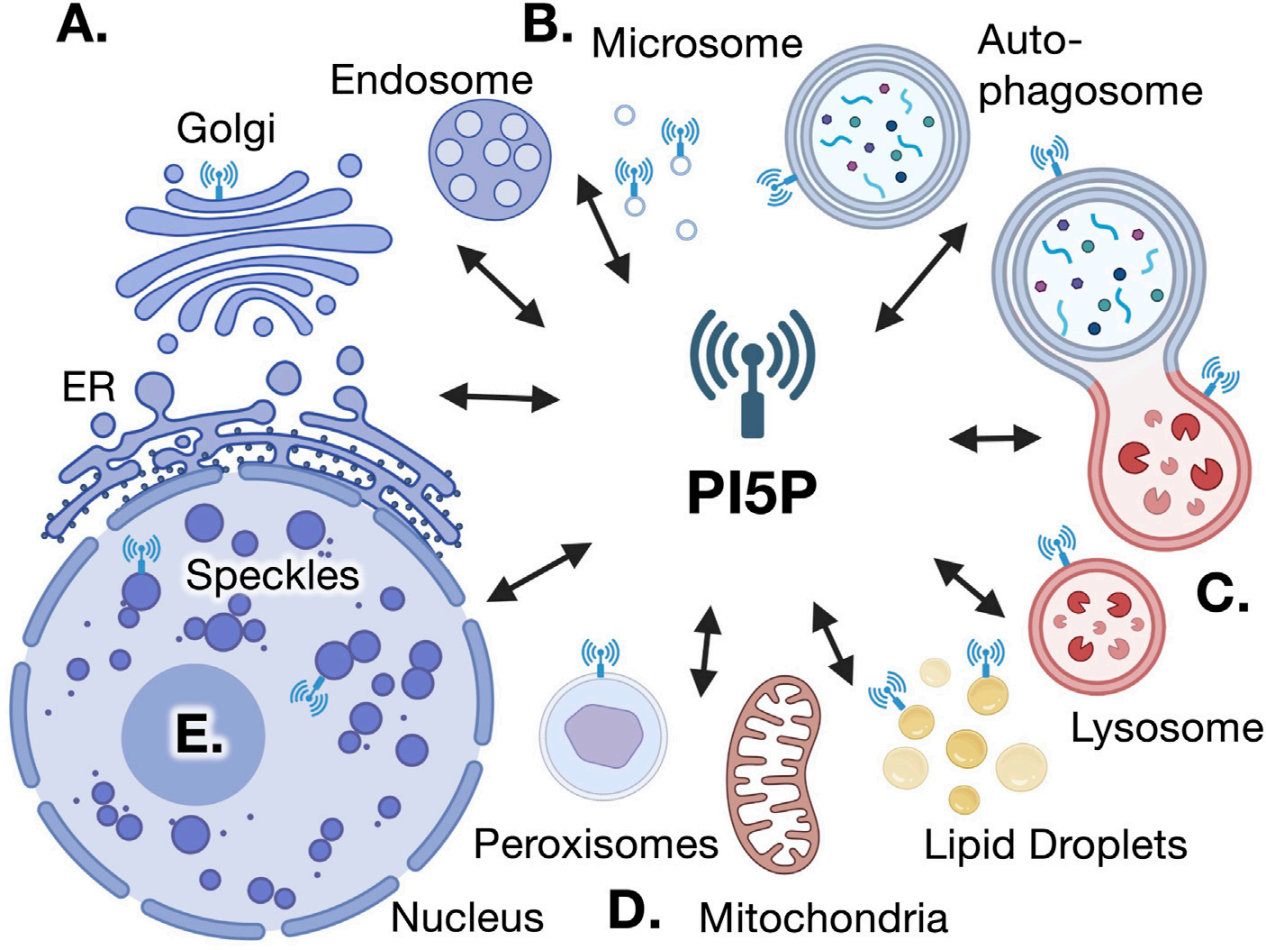
Putative sites of PI5P localization and action. Several lines of evidence support the existence of PI5P within various intracellular compartments, which reciprocally communicate various aspects of cell physiology. **(A).** PI5P was found by direct HPLC measurements in cellular fractions that colocalize with Golgi vesicles, also co-localized with PIP4Kγ (Sarkes and Rameh, 2010). **(B).** HPLC measurements of PI5P at light microsomal vesicles have been co-localized with PIP4K sites (Sarkes and Rameh, 2010). **(C).** Localization of the PI(4,5)P_2_ 4-phosphatase TMEM to lysosomes and endosome is expected to generate PI5P at these signaling interfaces (Zou, et al., 2007; Hashimoto, et al., 2018). The presence of GFP-PHD and PIP4Ks around autophagosomes and autolysosomes also suggest the presence of PI5P in these organelles (Vicinanza et al., 2015; Lundquist et al., 2018). **(D).** PI(4,5)P_2_ synthesis at the peroxisome occurs in a PIP4K-dependent manner, strongly suggesting the presence of PI5P in this organelle (Hu et al., 2018; Ravi et al., 2021) and the use of ING2-PHD probes indicate that PI5P may be present surrounding lipid droplets (Akil et al., 2016). **(E).** Direct mass assay measurements have detected PI5P in the nucleus (Jones, et al., 2018). Together, these data suggest PI5P is not evenly distributed across all cellular membranes, suggesting spatially separated and specific functions for PI5P in various cellular compartments.

One detail often missed from this story is that PI5P is not the only substrate for PIP4Ks. Although PIP4Ks use monophosphorylated forms of phosphoinositides very specifically, and are unable to phosphorylate PI4P (the 4 position is already phosphorylated), PIP4Ks can phosphorylate PI3P to generate PI(3,4)P_2_ (Rameh et al., 1997). Whether mammalian PIP4Ks phosphorylate PI3P in cells is still unclear, however, flies with loss-of-function dPIP4K have increased PI3P in addition to PI5P, suggesting that dPIP4K can indeed regulate the cellular levels of both lipids (Ghosh et al., 2023). This often-overlooked detail may cause scientists to misinterpret data which relies on PIP4K, and to make assumptions on the levels and biological activities of PI5P. For example, the enzymatic assay that is often used to measure the mass of cellular PI5P relies on PIP4K specifically phosphorylating PI5P (Morris et al., 2000). If the nature of the product generated during this assay is not carefully confirmed by HPLC, as the original article shows, those using this method may indeed be measuring a combination of PI5P and PI3P, and thus make inaccurate conclusions. Adding to this problem is that many PI5P-binding modules lack sufficient specificity to distinguish PI5P from more abundant monophosphorylated phosphoinositides, confounding interpretation of the quantity and subcellular location of PI5P. Below, we discuss the advances in our understanding of PI5P function in cells (see Figure 1), in the context of these important caveats.

## 2 Pathways for PI5P generation and depletion

When the PI5P-dependent pathway for PI(4,5)P_2_ synthesis was first discovered, it was assumed that this pathway only contributes to a small fraction of the total cellular PI(4,5)P_2_, based on the much lower abundance of PI5P as compared to PI4P. Thus, it was proposed that PI5P could be a signaling molecule whose levels are controlled by PIP4Ks and/or an intermediate in the synthesis of a very specific subcellular pool of PI(4,5)P_2_. To better understand these potential roles for PI5P, it is critical to establish the subcellular localization of PI5P (discussed below) and the enzymes involved in its generation and depletion. Although it is clear that PIP4Ks consume cellular PI5P by converting it into PI(4,5)P_2_, the pathway(s) for PI5P synthesis in cells is/are still subject of much debate. Inhibiting PIKfyve, a 5-kinase that is known for generating PI(3,5)P_2_, has been reported by numerous investigators to decrease (but not completely eliminate) cellular PI5P, indicating that PIKfyve is partially responsible for the synthesis of PI5P (Sbrissa et al., 2012; Er et al., 2013). The controversy however is whether PI5P is directly generated from the phosphorylation of PI (phosphatidylinositol) by PIKfyve (Shisheva et al., 2015) or by the dephosphorylation of PI(3,5)P_2_ by a 3-phosphatase (Hasegawa et al., 2017), a reaction that is accomplished by the myotubularin family of lipid phosphatases (Schaletzky et al., 2003; Tronchère et al., 2004), or some combination of both. The issue is further complicated by the fact that PIKfyve is associated with a PI3Kinase activity (Sbrissa et al., 1999). Thus, it is possible that the PIKfyve/PI3K complex generates PI(3,5)P_2_ through one or two different routes, distinguished by the 5′-phosphate being added first or last.

Five years after the discovery of PI5P, Bernard Payrastre’s group reported for the first time, that the enteropathogen *Shigella flexneri* generates extremely high levels of cellular PI5P upon bacterial invasion (Niebuhr et al., 2002). The rapid increase in cellular PI5P was due to injection of the phosphatase IpgD through the type III secretory system. IpgD was found to be a PI(4,5)P_2_ phosphatase that specifically removes the 4-phosphate to generate PI5P during cell invasion, suggesting an important role for PI5P in this process. As discussed below, the role of PI5P in the invasion process has not been fully determined and is difficult to separate from the effects of PI(4,5)P_2_ loss or the observed increase in PI(3,4,5)P_3_, a cell survival signal produced in response to bacterial invasion. Nevertheless, these findings established precedence to search the mammalian genome for analogous phosphatases that could potentially generate PI5P through dephosphorylation of PI(4,5)P_2_. In fact, the group led by Philip Majerus identified two mammalian PI(4,5)P_2_ 4-phosphatases that can generate PI5P *in vitro* (Ungewickell et al., 2005). These enzymes are referred to as type I and II PI(4,5)P_2_ 4-phosphatases, TMEM55A and TMEM55B or PIP4P1 and PIP4P2. Whether these phosphatases can generate PI5P in cells remains poorly studied (Zou et al., 2007).

The enzymes directly involved in PI5P synthesis, PIKfyve and TMEMs, have been shown to localize to endosomes and to the lysosome (Zou et al., 2007; Hasegawa et al., 2017; Hashimoto et al., 2018). These organelles are likely to contain all three proposed immediate PI5P precursors, i.e., the low abundance PI(3,5)P_2_ as well as the high abundance PI and PI(4,5)P_2_. However, it is important to note that the immediate precursor of PI5P could be short lived, and thus undetectable, due to the concerted action of kinases and phosphatases, as has been proposed for the synthesis of PI(3,4,5)P_3_ under IQGAP scaffolding (Choi et al., 2016; Rameh and Mackey, 2016).

In summary, the only established pathway for PI5P synthesis in living mammalian cells is through PIKfyve and the only pathway for PI5P depletion is through PIP4K, however we are quick to acknowledge that other pathways are likely to exist, and more data are required to fully characterize the pathways for cellular PI5P regulation in mammalian cells.

## 3 PI5P as a signaling lipid in stress and growth factor responses

Shortly after PI5P was discovered, Robin Irvine’s group developed a strategy for measuring PI5P enzymatically using PIP4Kα to convert the PI5P present in cellular phosphoinositide extracts into PI(4,5)P_2_ which can be detected by either antibodies or isotope tracing (Morris et al., 2000). Using this mass assay, the group discovered that PI5P levels increase in platelets in response to thrombin stimulation (Morris et al., 2000). The same assay led to the discovery that PI5P increases in response to cellular stress, including oxidative stress and DNA damaging agents (Jones et al., 2006). Using measurements of isotope-labeled phosphoinositides isolated from cells and resolved by HPLC, we calculated that cellular PI5P levels are within 1%–4% of the levels of PI4P, depending on the cell type (Sarkes and Rameh, 2010). Although PI5P is constitutively present in most cells, its levels can be acutely (within minutes) stimulated not only by stress signals but also by growth factors, in particular insulin (Sbrissa et al., 2004; Sarkes and Rameh, 2010; Jones et al., 2013) and FGF (Viaud et al., 2014), reaching levels comparable to that of PI3P (Sarkes and Rameh, 2010). How PI5P stimulation contributes to growth factor signaling has not been fully resolved, but may involve activation of Akt, as suggested by a sharp reduction in Akt activation in cells overexpressing catalytically active PIP4Ks (Carricaburu et al., 2003). Three mechanisms were proposed to explain the PI5P-dependent activation of Akt: a) PI(3,4,5)P_3_ synthesis through PI3K activation (Pendaries et al., 2006); b) PI(3,4,5)P_3_ stability through phosphatase inhibition (Carricaburu et al., 2003); c) phospho-Akt stability through inhibition of PP2A phosphatase (Ramel et al., 2009). Direct putative targets of PI5P in the Akt pathway have not been identified. In the context of growth factor signaling, PI5P was also shown to play a critical role in actin remodeling (Sbrissa et al., 2004). Under physiological or pathological tyrosine kinase signaling, PI5P-dependent actin remodeling is thought to involve direct interaction and activation of TIAM1 (see Table 1), a nucleotide exchange factor for Rac1, demonstrating a potential role for PI5P in cell migration and tumor invasion (Viaud et al., 2014). It is important to note that PI3P can also bind and activate TIAM1 (see Table 1).

**TABLE 1:** Putative PI5P targets. Binding to additional phosphoinositides show a gross estimate based on the data provided by each paper, with = representing no difference, > representing small difference and >>> representing strong difference.

Putative PI5P-binding protein	Binding to additional PIPs	References
ING2-PHD domain	PI5P > PI3P > PI4P	Gozani et al. (2003)
Dok1 and Dok2 PH domains	PI5P = PI4P > PI3P	Guittard et al. (2009)
Dok5 PH domain	PI5P > PI4P > PI3P	Guittard et al. (2010)
IRF3	Only PI5P tested	Kawasaki et al. (2013)
WIPI2B	PI5P = PI3P>>>PI	Vicinanza et al. (2015)
Tiam1 PH domain	PI5P > PI3P > PI4P	Viaud et al. (2014)
TAF3-PHD	PI5P = PI3P > PI(4,5)P_2_	Stijf-Bultsma et al. (2015)
CXXC1, NSD1, ING3-PHD	PI5P = PI(4,5)P_2_ >PI3P	
Septin 9	PI5P = PI3P = PI4P	Akil et al. (2016)

When mass assay or HPLC techniques were used to analyze the phosphoinositide composition of subcellular fractions, PI5P was found in several fractions associated with different membrane organelles, but it was particularly abundant in light microsomal fractions (Sarkes and Rameh, 2010) and in the nucleus (Jones et al., 2006). Nuclear PI5P increases in cells challenged with oxidative and DNA damaging stressors due in part to phosphorylation and inhibition of PIP4Kβ by p38 MAP kinase, a stress induced kinase (Jones et al., 2006). FGF-stimulated PI5P was largely dependent on PIKfyve activity (Viaud et al., 2014). In the case of peroxide-induced PI5P, the mechanism for PI5P synthesis is unknown but thought to involve activation of a PI(4,5)P_2_ phosphatase, rather than PIKfyve (Jones et al., 2013). Thus, the origin and localization of stress-induced PI5P likely depends on the signal and the enzymes involved, and may provide clues as to how peroxides in the tumor microenvironment and other cell stressors contribute to cancer (Hockenbery et al., 1993; Keune et al., 2012). Activation of PI5P in the context of stress signals has been linked to activation of apoptotic signals (Gozani et al., 2003), however clearly more data is required to fully describe the role of PI5P in stress and growth factor signaling.

## 4 PI5P and pathogen invasion

Cellular invasion by the enteropathogen *Shigella flexneri* results in rapid and dramatic generation of cellular PI5P at the site of invasion through the action of the lipid phosphatase IpgD (Niebuhr et al., 2002). This is also true for another enteropathogen, *Salmonella*, which expresses the virulence factor SigD that, just like IpgD, dephosphorylates PI(4,5)P_2_ to generate PI5P (Mason et al., 2007). Both IpgD and SigD significantly contribute to the invasion process by causing cytoskeleton breakdown and membrane blebbing (Niebuhr et al., 2002; Terebiznik et al., 2002). PI(4,5)P_2_ consumption is likely to play critical roles in the cytoskeleton and membrane changes at the site of entry, nonetheless, it is difficult to dissect the role of PI(4,5)P_2_ disappearance from the role of PI5P synthesis. One intriguing hypothesis may be that generation of PI5P during PI(4,5)P_2_ breakdown is evolutionarily advantageous to the pathogen, as opposed to generation of PI4P. Interestingly, PI5P generated from *Shigella* was shown to aid the bacteria in the ability to evade the host immune system by inhibiting stress responses initiated by infected cells (Puhar et al., 2013). When pathogens infect epithelial cells, they respond by releasing ATP into the extracellular environment through connexin hemichannels as a signal to initiate inflammatory responses. Expression of catalytic-active exogenous IpgD inhibited calcium depletion-induced hemichannel opening, whereas expression of catalytic-dead IpgD or PI(4,5)P_2_ 5-phosphatase (Inp54p) that generate PI4P instead, did not (Puhar et al., 2013). In these studies, exogenous PI5P on its own was able to inhibit the connexin hemichannels and ATP release, whereas PI3P or PI4P had no effect. These results suggest a role for PI5P in the virulence of *Shigella flexneri*. Another role for PI5P during pathogen invasion was suggested by findings that PI5P is likely to contribute to increase in IpgD-induced Akt activation (Carricaburu et al., 2003; Pendaries et al., 2006; Ramel et al., 2009;), a cell survival signal that may aid the bacteria by keeping the host cells alive during the invasion process. Consistent with this idea, IpgD-induced PI5P localized to the endosome prevented EGF receptor degradation and promoted its activation, thereby contributing to increased Akt signaling (Ramel et al., 2011). More recently, IpgD was shown to facilitate bacterial dissemination from cell to cell by helping resolve membrane protrusions into double membrane vacuoles that carry bacteria from one cell to another (Köseoğlu et al., 2022). Furthermore, IpgD catalytic activity was shown to help the bacterium evade the immune system by inhibiting T-cell migration (Konradt et al., 2011) and to modulate signaling downstream of the T-cell receptor that leads to interleukin-2 expression (Guittard et al., 2009; 2010). In these studies, the T-cell receptor downstream kinases Dok-1, 2, 4 and 5 were shown to bind PI5P, but only Dok-5 showed some level of specificity (see Table 1), although further affinity measurements will be necessary to validate this as a PI5P probe.

Although PI5P generated by bacteria aids the pathogen in evading the immune system, in the case of viral infection, the opposite occurs. Studies have shown a role for PI5P generated during viral infection, in particular by the Newcastle disease virus, in triggering antiviral innate immunity by the host cells through induction of interferon secretion (Kawasaki et al., 2013). The investigators suggested a model in which direct interaction between PI5P and interferon regulatory factor-3 (IRF3, see Table 1), causes changes in protein conformation that makes IRF3 accessible to phosphorylation by TBK1, a kinase known to activate IRF3. The authors propose that synthetic short-chain PI5P could be used as an adjuvant by stimulating cytokine production (Kawasaki et al., 2013). It is important to note that increased levels of PI5P after viral infection were very mild (50% over basal) as compared to the increases elicited by the bacterial virulence factors IpgD and SigD. Additionally, measurements of PI5P in this study could be overestimated due to contamination with PI3P, a caveat of the mass assay that can only be resolved by HPLC analysis of the product obtained (see above).

## 5 PI5P in intracellular membrane dynamics

Phosphoinositides have been known to play critical roles in membrane trafficking events by recruiting proteins involved in vesicle transport to specific subcellular membrane compartments, reviewed by (Di Paolo and De Camilli, 2006). Thus, it is likely that PI5P participates in these cellular processes as well. In fact, using *Drosophila* photoreceptor as a model system, the group led by Dr. Padinjat described a PIP4K-dependent defect in clathrin-mediated endocytosis (Kamalesh et al., 2017). In mammalian cells, the following intracellular membrane process with major implications for inter-organelle communication and metabolic homeostasis have been linked to the PI5P pathway for PI(4,5)P_2_ synthesis: autophagy, peroxisome cholesterol accumulation, lipid storage and degradation and mTORC1 regulation. The roles of PI5P in all these processes are expanded on in the below sections.

## 6 Autophagy

Rubinsztein and collaborators directly addressed the role of PI5P in autophagy using a variety of approaches that when combined led them to propose that PI5P is involved in autophagosome biogenesis, a function that is normally performed by PI3P (Vicinanza et al., 2015). The authors proposed that during glucose-deprivation PI5P serves as an alternative lipid to signal for recruitment of proteins associated with autophagy initiation, namely, WD-repeat associate with PI (WIPI2) through direct binding to PI5P (see Table 1). Following that discovery, the group led by Brooke Emerling suggested that in cells with double knockdown/knockout of PIP4Kα and PIP4Kβ, the increases in autophagosome are also accompanied by a decrease in autophagic flux, implying that either PI5P accumulation is detrimental to fusion of autophagosome vesicles to the lysosome or the lack of PI5P-derived PI(4,5)P_2_ at these organelles impairs fusion (Lundquist et al., 2018). Interestingly, the decreased autophagic flux observed in the PIP4K double knockout cells is accompanied by a complex phenotype which involves generalized metabolic imbalances, mTORC1 signaling defects and increased transcription of genes involved in autophagosome or lysosome biology (Lundquist et al., 2018). Remarkably, they observed increased apoptosis in liver and growth defects in MEFs from the PIP4Ks double knockouts. Thus, it is clear that the integrity of the PI5P pathway for PI(4,5)P_2_ synthesis is critical for cell survival and growth. In particular, cancer cells seem to rely on this pathway and investigators have proposed to target these lipid kinases in the potential treatment of p53-null cancers (Emerling et al., 2013). Given the complexity of the phenotypes observed in these cells, it is hard to attribute cause and effect to each of these perturbations that will allow us to infer direct roles for PI5P and/or PI5P-derived PI(4,5)P_2_ on these outcomes. For example, it is still unclear if PIP4K catalytic activity is important for re-establishing flux through autophagy, which is a particularly relevant piece of the puzzle, given that PIP4K can function in a kinase-independent manner to chaperone and negatively regulate PIP5Ks (Wang et al., 2019). Furthermore, in *Drosophila*, the effects of dPIP4K loss-of-function on cell size and autophagy have been recently attributed to the ability of this kinase to regulate cellular PI3P levels rather than PI5P (Ghosh et al., 2023).

## 7 Peroxisome function

A direct role for PI5P-derived PI(4,5)P_2_ synthesis in peroxisome function and cholesterol homeostasis was first proposed by a group led by Song and others (Hu et al., 2018). They found that PI(4,5)P_2_ generated by PIP4Kα on the surface of peroxisomes promotes lysosome/peroxisome fusion and transport of cholesterol from the lysosome to peroxisome for further metabolism. When PIP4Kα was knocked down from HEK 293 cells, there was a reduction in PI(4,5)P_2_ in purified peroxisomes, as measured by dot blots using anti-PI(4,5)P_2_ antibody. The authors had previously reported that PI(4,5)P_2_ is required for peroxisomal/lysosomal contacts, which are important for hand-off of cholesterol and perhaps other lipids (see below) from one organelle to the other. As a result of PIP4K knockdown or knockout, they observed a decrease in peroxisome/lysosome contacts as measured by subcellular localization and biochemical pulldown assays, and a dramatic accumulation of cholesterol in lysosomes of cells with reduced PIP4Kα. This was further confirmed using *in vitro* assays with purified peroxisome and lysosome, which allowed them to rule out any secondary effects of PIP4K knockdown (Hu et al., 2018). Interestingly, knockdown of PIP4Kα, but not PIP5K, caused the cholesterol accumulation in cells, which indicates that this is indeed a function of PI5P-dependent PI(4,5)P_2_ synthesis on peroxisomes rather than PI4P-dependent PI(4,5)P_2_, which is catalyzed by PIP5Ks. The use of kinase-dead PIP4Kα would help confirm this.

The Emerling group expanded these findings to demonstrate that peroxisomal function is impaired in the PIP4Kα/PIP4kβ double knockout MEFs, possibly due to impaired transfer of lipids from lipid droplets to peroxisomes, suggested by the decreased co-localization of these two organelles upon challenge with lipid peroxidation agents (Ravi et al., 2021). Using immunofluorescence, they showed loss of PI(4,5)P_2_ co-localization with peroxisomes in the double knockout MEFs, similar to work in other labs (Hu et al., 2018). Importantly, expression of exogenous wild-type PIP4Kα in 293A cells rescued the co-localization of these two organelles, whereas the kinase-dead mutant did not (Ravi et al., 2021), indicating that the PI(4,5)P_2_ detected near peroxisomes is dependent on PI5P. Although peroxisome number was not affected by PIP4K double knockout, the expression of genes related to peroxisomal function was impaired, including the expression of enzymes involved in ß-oxidation and ROS detoxification, important peroxisomal functions. In association with peroxisome impairment, they observed a remarkable decrease in mitochondrial function, including changes in morphology, dramatically reduced mitochondrial membrane potential, impaired respiratory capacity, and lower ATP levels (Ravi et al., 2021). The PIP4K double knockout cells had significantly impaired proliferation and/or survival, especially under complete and prolonged glucose deprivation (16 h) or under supplementation with long-chain fatty acids. The complex metabolic and transcriptional alterations observed in these cells make it difficult to determine which phenotypes are directly related to depletion of PI(4,5)P_2_ vs. the predicted increase in PI5P. Nonetheless, these studies clearly suggest the PI5P pathway for PI(4,5)P_2_ synthesis plays a role in the maintenance of metabolic homeostasis, possibly through multiple mechanisms related to the crosstalk between membrane organelles. Exploring the metabolic vulnerability of cells lacking PIP4K activity is a major goal of drug development programs targeting PIP4K in the treatment of cancers and potentially other metabolic diseases (Arora et al., 2022).

## 8 Lipid droplets

Changes in lipid homeostasis is a common phenotype observed by various researchers after perturbations in the PI5P pathway for PI(4,5)P_2_ synthesis. The group led by Gassama-Diagne reported in 2015 that cellular PI5P manipulations resulted in changes in the size of lipid droplets induced by hepatitis C virus (HCV) in hepatocytes (Akil et al., 2016). Using exogenous PI5P or IpgD-induced PI5P, the investigators showed a dramatic increase in lipid droplet size, which the authors attributed to direct binding of PI5P to Septin 9 (see Table 1), a member of a large family of GTP-binding proteins associated with cytoskeleton and with known roles in vesicle trafficking events (Fung et al., 2014). Although PI3P and PI4P were also able to bind to septin-9 *in vitro*, albeit to a lower extent, and induce increase in lipid droplet size in cells, PI5P had the strongest effect with a 3-fold increase in lipid droplet size. IpgD expression increased PI5P and induced a 5-fold increase in lipid droplet size, whereas treatment with the PIKfyve inhibitor YM201636 decreased cellular PI5P (as measured through the PHD-probe) and decreased the size of septin9-induced lipid droplet. The authors concluded that PI5P cooperates with septin9 to promote lipid droplet expansion during HCV infection (Akil et al., 2016). Interestingly, hepatocytes from mouse with double knockout of PIP4Kα and PIP4Kβ, which are predicted to have increased PI5P, also showed increase in lipid droplet number and size (as measured by oil red staining) and liver triglyceride content, with similar phenotype seen in the double knockout MEFs (Lundquist et al., 2018). Likewise, in HEK293 cells with knockdown or knockout of PIP4Kα, an accumulation of cellular cholesterol was observed (Hu et al., 2018). Thus, high levels of PI5P correlates with lipid accumulation in several different cell lines. It is tempting to speculate that the role of the PI5P pathway for PI(4,5)P_2_ synthesis in lipid homeostasis is two-fold: i) to promote lipid droplet expansion, a phenotype likely attributed to PI5P, and ii) to facilitate organelle contacts that deliver cholesterol and very long-chain fatty acids to the peroxisome for breakdown, phenotypes attributed to PI5P-dependent PI(4,5)P_2_ synthesis. However, these potential mechanisms will require more data to differentiate and apply the appropriate weight to their roles in lipid droplet biology.

## 9 Nutrient sensing and homeostasis

Perturbations in the PI5P-pathway for PI(4,5)P_2_ synthesis are intricately connected to nutrient sensing. For example, PI5P was proposed to replace the role for PI3P as an initiator of autophagosome biogenesis upon glucose starvation (Vicinanza et al., 2015). The phenotypes observed in the PIP4K α/β double knockout MEFs and liver are exacerbated by either glucose starvation, serum starvation or by lipid overload (Lundquist et al., 2018). In addition, PIP4kβ was proposed to be a GTP sensor (Sumita et al., 2016). PIP4Ks have also been shown to regulate signaling through mTORC1, a nutrient-sensing kinase and major hub for integrating nutrient signals to cellular metabolic reprograming (Mackey et al., 2014). Using different cell-based systems and model organisms, several groups have demonstrated decreased mTORC1 signaling upon suppression of PIP4Ks (Gupta et al., 2013; Mackey et al., 2014; Lundquist et al., 2018) or pharmacological inhibition (Chen et al., 2021). In fact, the gamma isoform of PIP4K was shown to be a direct substrate for mTORC1 and a positive regulator of mTORC1 activity (Mackey et al., 2014). In one report, the ability of PIP4Kγ to increase mTORC1 activity was enhanced by the lipid ceramide (Zhang et al., 2020). PIP4Kγ has only low levels of kinase activity towards PI5P when tested *in vitro*, and PIP4Kγ ability to increase mTORC1 signaling does not rely on PIP4Kγ catalytic integrity (Mackey et al., 2014). However, PIP4K isoforms can heterodimerize with each other (Clarke et al., 2008; Bultsma et al., 2010; Wang et al., 2010; Clarke and Irvine, 2012). Interestingly, knockdown of PIP4Kγ was shown to result in more robust increases in PI5P than knockdown of the other two isoforms individually (Sarkes and Rameh, 2010). Thus, the ability of PIP4Kγ to increase mTORC1 may depend on the ability of PIP4Kγ to heterodimerize and act as a chaperone for the α and β isoforms. Surprisingly, knockout of PIP4Kγ in mice resulted in the opposite phenotype as that observed in cell lines, with the knockout mice having increased mTORC1 activity (Shim et al., 2016), suggesting the role of PIP4Ks in mTORC1 regulation is likely complex and is subject to multiple modes of feedback regulation (Mackey et al., 2014).

Given that mTORC1 dysregulation was reported in multiple publications from multiple organisms and cell types, it will be important to determine the mechanisms by which the PI5P pathway for PI(4,5)P_2_ synthesis regulates this nutrient-sensing complex. Are the metabolic deficiencies observed with PIP4K suppression causing mTORC1 inhibition or a consequence of mTORC1 inhibition? Answering this question is of course complicated by the reciprocal connection between mTORC1 activation and metabolism, e.g., mTORC1 is regulated by mitochondrial metabolism that generates ATP, but mTORC1 is simultaneously a positive regulator of mitochondrial biogenesis and function. In fact, inhibition of mTORC1 by itself recapitulated some of the PIP4K α/β double knockout phenotype related to changes in gene expression (Ravi et al., 2021). It is tempting to speculate that under nutrient starvation, heterodimers of PIP4Kα/β and PIP4Kγ could function to maintain basal mTORC1 signaling as has been proposed (Mackey et al., 2014), and that this is critical to keep proper metabolic homeostasis during nutrient scarcity. Thus, in cells lacking PIP4Kα and β, energy stress and decreased basal mTORC1 may engage in a negative feedback loop that results in metabolic collapse and cellular demise (Emerling et al., 2013; Lundquist et al., 2018; Ravi et al., 2021).

## 10 Subcellular localization of PI5P and nuclear PI5P

As in Section 1 mentioned earlier, studies of PI5P have been hampered by the lack of PI5P-specific probes. The PHD finger of the chromatin associated protein ING2 has been widely used, in the form of a 3x tandem repeat fused to GFP, as a probe for examining the fluctuations and localizations of cellular PI5P (Gozani et al., 2003). However, given its ability to bind to PI3P and PI5P with similar affinities (See Table 1), interpretation of the GFP-PHD signal can be complex. However, Sarkes et al. used a classic biochemical subcellular fractionation and HPLC analysis to track PI5P in pancreatic beta cells and HeLa cells (Sarkes and Rameh, 2010). This approach showed an enrichment in PI5P in light microsomal vesicles and vesicles associated with the Golgi complex, a distribution that overlapped with the pattern for PIP4Kγ. This distribution is consistent with a role for PI5P in intra-organelle communication, although this hypothesis remains to be directly tested. As described above, fluorescence probe-based tracing led to the detection of lipid droplets and autophagosomes as potential sites for PI5P subcellular localization and peroxisomes as potential sites for PI5P-dependent PI(4,5)P_2_ synthesis (Vicinanza et al., 2015; Akil et al., 2016; Hu et al., 2018). In addition, PI5P has been detected inside the nucleus, based on subcellular fractionation and enzymatic detection as described above (Jones et al., 2006). Increased PI5P in the nucleus was shown to activate transcription through direct binding to the transcription factor TAF3 (Stijf-Bultsma et al., 2015). Work from the lab of Wolfgang Fischle suggested PI5P allosterically activates the epigenetic regulator UHRF1, with PI5P inducing a state in UHRF1 that interacts more with methylated histones (H3K9me3) (Gelato et al., 2014; Mandal et al., 2022). More recently, PI5P exogenously added to cells was shown to promote the degradation of UHRF1 (Poli et al., 2023). PI5P has also been previously shown to promote ubiquitin-dependent protein degradation (Bunce et al., 2008). As measured using the PHD domain of ING2, PI5P localization in the nucleus is polarized and that PI5P polarity is lost upon knockdown of PIP4Kβ (Poli et al., 2023). The investigators show that when cells are plated in soft substrates, PIP4Kß protein is degraded, which presumably results in PI5P increase in the nucleus (not measured) and UHRF1 degradation, ultimately leading to the egress of the mechanosensor and transcriptional regulator YAP from the nucleus to the cytosol. The authors proposed that PIP4Kβ functions as a mechanosensor to induce gene transcription through regulation of PI5P/UHRF1/YAP (Poli et al., 2023). For a more comprehensive review on the nuclear functions of PI5P we refer the reader to another review (Poli et al., 2019).

## 11 Conclusions and future studies

Since its discovery in 1997, PI5P has been linked to important cellular functions such as actin remodeling, vesicle trafficking, metabolic homeostasis and apoptosis/cell survival in the context of physiological or pathological signaling. Thus, the search for pharmacological agents that inhibit PIP4K activity and thereby increase cellular PI5P is justified and holds potential benefits in the treatment of cancers and metabolic diseases. Future research on how PI5P signaling differs from signaling by PI3P or PI4P awaits development of better tools to dissect the spatiotemporal niche for PI5P. Many questions remain, such as can PI5P generate cellular responses that other signaling phosphoinositides cannot? Is PI5P signaling temporally or spatially coupled to PI(4,5)P_2_ generation/disappearance, or related to PI(3,5)P_2_ consumption? One clue is the notion that PI5P-dependent PI(4,5)P_2_ synthesis is only present in complex multicellular organisms. Thus, PI5P function likely participates in perhaps more complex, specialized cell regulatory tasks specific to more complex organisms. The next 25 years will hopefully reveal the answers we’ve been looking for.
